# WHO policy development processes for a new vaccine: case study of malaria vaccines

**DOI:** 10.1186/1475-2875-9-182

**Published:** 2010-06-24

**Authors:** Julie Milstien, Vicky Cárdenas, James Cheyne, Alan Brooks

**Affiliations:** 1 University of Maryland School of Medicine, 3 bis rue des Coronilles Résidence Parc de Clémentville Bâtiment C, 34070 Montpellier, France; 2 PATH Malaria Vaccine Initiative, 7500 Old Georgetown Road, #1200 Bethesda, MD 20814, USA; 3 PATH, 13 Chemin du Levant, 01210 Ferney-Voltaire, France

## Abstract

**Background:**

Recommendations from the World Health Organization (WHO) are crucial to inform developing country decisions to use, or not, a new intervention. This article analysed the WHO policy development process to predict its course for a malaria vaccine.

**Methods:**

The decision-making processes for one malaria intervention and four vaccines were classified through (1) consultations with staff and expert advisors to WHO's Global Malaria Programme (GMP) and Immunization, Vaccines and Biologicals Department (IVB); (2) analysis of the procedures and recommendations of the major policy-making bodies of these groups; (3) interviews with staff of partnerships working toward new vaccine availability; and (4) review and analyses of evidence informing key policy decisions.

**Case description:**

WHO policy formulation related to use of intermittent preventive treatment in infancy (IPTi) and the following vaccine interventions: *Haemophilus influenzae *type b conjugate vaccine (Hib), pneumococcal conjugate vaccine (PCV), rotavirus vaccine (RV), and human papillomavirus vaccine (HPV), five interventions which had relatively recently been through systematic WHO policy development processes as currently constituted, was analysed. Required information was categorized in three areas defined by a recent WHO publication on development of guidelines: safety and efficacy in relevant populations, implications for costs and population health, and localization of data to specific epidemiological situations.

**Discussion and evaluation:**

Data needs for a malaria vaccine include safety; the demonstration of efficacy in a range of epidemiological settings in the context of other malaria prevention interventions; and information on potential rebound in which disease increases subsequent to the intervention. In addition, a malaria vaccine would require attention to additional factors, such as costs and cost-effectiveness, supply and demand, impact of use on other interventions, and distribution issues.

**Conclusions:**

Although policy issues may be more complex for future vaccines, the lead-time between the date of product regulatory approval and a recommendation for its use in developing countries is decreasing. This study presents approaches to define in advance core data needs to support evidence-based decisions, to further decrease this lead-time, accelerating the availability of a malaria vaccine. Specific policy areas for which information should be collected are defined, including studying its use within the context of other malaria interventions.

## Background

It is a priority for both the PATH Malaria Vaccine Initiative (MVI) and the World Health Organization (WHO) to determine the most appropriate role for a future malaria vaccine in health systems of countries at risk for malaria mortality and morbidity. The most advanced first-generation malaria vaccine candidate has shown promising results [1-4]. If all goes well, general implementation of RTS,S for infants six to 12 weeks of age is possible within five years or so. The vaccine could be available for targeted use among young children five to 17 months old as early as 2013. Other malaria vaccines are anticipated some years later. It is imperative to define how to most effectively develop malaria vaccine implementation policies at the national, regional, and global levels well in advance of the first vaccine.

MVI previously developed a policy pathway for a malaria vaccine in consultation with WHO and other partners. One early step in MVI's policy pathway is to identify the data--and the timing--required for a WHO policy position on malaria vaccines. This information can then guide MVI and its partners to develop the required data at the correct time over the coming years.

Ultimately, this analysis can streamline the process of establishing policy recommendations, leading to more rapid availability of an appropriate product than has been seen previously. The information included in this analysis could be useful to guide other groups developing new treatment or preventive interventions for the developing world. Cutting time off the policy-making process will ultimately mean that life-saving interventions can get to those in need much sooner than has been seen historically.

## Methods

### Selection of interventions to study

One malaria intervention and four vaccine interventions were analysed in detail to understand the data and their availability to WHO policymaking bodies. The malaria intervention was delivery of anti-malarial drugs to children during immunization sessions - intermittent preventive treatment in infancy (IPTi). The vaccine interventions were *Haemophilus influenzae *type b conjugate vaccine (Hib), pneumococcal conjugate vaccine (PCV), rotavirus vaccine (RV), and human papillomavirus vaccine (HPV). These interventions were selected as they had relatively recently been through systematic WHO policy development processes as currently constituted.

### Study methodology

The analysis was conducted by interviewing primary contacts (see acknowledgements) and reviewing documents and websites related to the five interventions described above. The authors adhered to the PRISMA guidelines for improving the quality of reporting of systematic reviews and meta-analyses to the extent possible [5]. Because the aim of this paper is to describe and systematize the interpretation of WHO policy, the authors consulted individuals from those departments in WHO who contributed, or were relevant to, the decisions made by the groups in question for the five interventions analyzed. In addition selected non-WHO staff who led initiatives specifically supporting WHO policy development were identified and interviewed. In total 9 individuals were identified across the 5 interventions. The authors reviewed websites from IVB, the IVB Strategic Advisory Group of Experts (SAGE), GMP, Roll Back Malaria Partnership (RBM) and the IPTi Consortium. The authors reviewed every document indicated as reference documents for the relevant advisory groups as well as those recommended by the interviewees, a total of over 100 documents. Of these, the majority were excluded from the analysis as not reflecting key milestones on the policy process. Twenty-four documents which elaborated milestones on the policy process were retained. It is not possible to know the extent of, or the role played by, unrecognized individuals and/or undocumented decision-making.

### Case description

#### WHO policy development process

It is likely that two expert groups will consider future policy positions for a malaria vaccine: the Global Malaria Programme (GMP) Strategic Technical Advisory Group (STAG) and the Immunization, Vaccines and Biologicals Department (IVB) SAGE. The GMP Director has only recently established the STAG, charged with developing malaria policy recommendations, based on expert inputs [6]. Therefore, STAG's operating processes do not yet have the history of the corresponding group for vaccines. STAG is advisory ultimately to the Director-General of WHO. Three technical expert groups (TEGs), on chemotherapy of malaria, vector control, and economics and financing, report to STAG.

Within IVB, development of vaccine policy positions rests with SAGE [7,8]. SAGE has existed in its current configuration since November 2005 as the principal advisory group to the WHO Director-General for development of policy recommendations related to vaccines and immunization. It is charged with advising WHO on policies and strategies on vaccines and technologies, research and development, delivery of immunization, and their linkages with other health interventions for all vaccine preventable diseases, providing strategic advice rather than technical inputs. The deliberations of SAGE take into consideration the recommendations of two additional WHO expert groups, the Global Advisory Committee on Vaccine Safety (GACVS) and the Expert Committee on Biological Standardization (ECBS) [9].

WHO policy recommendations on vaccine use are expressed in WHO vaccine position papers published in the *Weekly Epidemiologic Record*. Since April 2006, these position papers have been fully discussed and endorsed by SAGE prior to publication; every effort is made to coordinate their publication as soon as possible after the relevant SAGE meeting.

SAGE may consider a matter at least three times in deciding whether or not to issue a policy recommendation. The first meeting is to inform SAGE members of potential new interventions on the horizon. The second meeting, which occurs when the potential intervention is close to the finish, is extremely important, as it provides a clear indication of the kinds of information SAGE would need to make a global recommendation (i.e., a recommendation for use applicable to all target populations). If all needed information is in place, the third meeting can be the decision-making session, whether a utilization recommendation or a more limited position is solicited. Since the SAGE process is dynamic and evolving, this description may not hold in all cases.

#### Classification of data: WHO Guidelines

In 2003, WHO published *Guidelines for WHO Guidelines *[10]. This publication emphasizes the use of systematic reviews for evidence of effects for a policy option (such as a treatment protocol or use of a preventive intervention) and of evidence-based dissemination and implementation strategies [11,12]. The *Guidelines *outline three categories of input in development of a policy option:

• Reviewing and reporting of efficacy and safety

• Considering the implications of adopting the policy positions in relation to costs and population health

• "Localizing" the guidelines to their settings and determining where the tradeoff of additional cost *vs *additional benefit will be set

Because these three *Guidelines *categories appear relevant to the analytical processes of the advisory groups considered, these categories have provided a framework to define the information available and data needs for policy decisions on the interventions considered in this analysis (See Table 1).

**Table 1:** Analysis of data available for SAGE vaccine decisions according to WHO Guidelines document criteria

Intervention	Hib	PCV	RV	HPV	IPTi
Year of licensing	1988	2000	2004	2006	NA

Safety and efficacy in relevant populations					

Industrialized countries only	<1998	1999	2004	2006	NA
Range of countries, missing information	1998	2003	2005	2007	2006
All data available	2006	2005	2009	2008	2009

Implications for costs and population health					

Impact on disease addressed	2005	2005	2004	2007	2004
Demand, supply, financing addressed	2006	2006	2005	2008	2009
All addressing including other public health	2006	2006	--	2009	2009

Localization of data					

Logistics issues addressed	NA	--	--	2007	2006
All delivery strategies addressed	2003	2003	2005	2008	2009
Successful demonstration project	2005	NA	2006	2008	2009

Date of global recommendation in WHO position paper	Nov 2006	Mar 2007	Aug 2007 (limited), Jun 2009	Jun 2009	Oct 2009

NA = not applicable; -- = not yet completed

#### Vaccine policy development

Table 1 summarizes the data available when policy positions were issued by the SAGE.

##### Haemophilus influenzae type b conjugate vaccine

In March 1998, WHO published the first position paper on Hib [13]. At that time safety and efficacy data were available both in some developing countries, such as Chile, the Gambia, and Uruguay [13], and in industrialized countries [14,15]. Efficacy was found to be at least 95 percent against invasive *Haemophilus influenzae *type b disease after three doses. There were no vaccine safety concerns. However, there was little quantification of disease burden outside of the industrialized world and most countries did not have a surveillance system sufficiently tuned to measure vaccine impact. The WHO position paper recommended the use of Hib in routine infant immunization programmes in view of its demonstrated efficacy and safety, but noted the lack of robust disease burden data in Asia and in the Newly Independent States.

In 2003 and again in 2005, SAGE reviewed disease burden data in Asia [16,17], and in 2005 recommended global implementation of Hib vaccination, unless there were robust epidemiological evidence of low disease burden, lack of benefit, or overwhelming impediments to implementation [17]. A new position paper on Hib was then published in November 2006 [18]. This paper gave a strong recommendation for Hib use, stating clearly that lack of local surveillance data should not delay vaccine introduction.

Thus, in summary, a global recommendation on Hib use was not made until 2006 for a vaccine that had been licensed 15 years previously, in 1988, and had been covered by a WHO Position Paper since 1998. The 2006 recommendation was made almost immediately following the presentation to SAGE of information on disease burden and efficacy in relevant target populations, and after supply issues had been addressed. The latter element indicated the concern of SAGE with information additional to safety and efficacy, as described in the *Guidelines *categories mentioned above.

Since that recommendation and other activities coincident with the establishment of the Hib Initiative, Hib uptake has increased considerably: from 40 countries in 1998 to 103 in 2007. By the end of 2009, it is projected that a total of 165 countries, including 61/72 of the world's poorest countries, will have introduced the vaccine [19,20].

##### Pneumococcal conjugate vaccine

The original WHO PCV position paper was published in April 2003 [21]. The paper reviewed data on safety and efficacy of the seven-valent conjugate vaccine in the United States (US) and other industrialized countries. It noted the vaccine was up to 97 percent effective against invasive disease caused by the serotypes in the vaccine, with no apparent safety concerns, that price would be an issue, and suggested that "the conjugate vaccine merits consideration for inclusion in national childhood immunization programmes."

In November 2005 [17], SAGE noted PCV safety and efficacy data in a wide variety of settings, including South Africa [22], and the Gambia [23]. In addition, SAGE recognized the supply and financing issues with PCV, produced in limited quantities by a monopoly supplier, and said, "a global recommendation made before resolution of funding and supply issues would leave vulnerabilities that have been experienced with the implementation of Hib." Again, this indicates attention to issues other than safety and efficacy.

The assembly of further data was accelerated by the naming of a SAGE working group set up to lay the groundwork for an evidence-based recommendation. More information on safety and efficacy in different settings became available [24], as well as cost-effectiveness data [25]. In November 2006, in response to this information, SAGE, satisfied with the information provided on supply, cost, and cost-effectiveness, gave a strong recommendation for use of the seven-valent conjugate vaccine [26]. These recommendations then formed part of the revised position paper on PCV, published in March 2007 [27].

Thus, for PCV, licensed in 2000, and covered by a WHO position paper since 2003, a global recommendation was made in 2007, a delay between licensure and recommendation of only seven years. This followed receipt not only of stronger data on safety and efficacy in all target populations, but also of information on prospects for addressing supply and financing issues.

Of the 26 countries having introduced PCV as of August 2008, none were low or lower middle income countries. Recently, Rwanda became the first low income country to introduce PCV [28,29].

##### Rotavirus vaccine

The original WHO position paper on RV appeared in 1999 [30] and gave recommendations on the use of the rhesus tetravalent reassortant vaccine developed by Wyeth and licensed in the USA in 1998. At that time there was little information demonstrating efficacy at the provided dose in developing countries, and additional efficacy trials were begun in Africa and Asia. When reports of intussusception appeared, WHO convened a meeting in 2000 to review the safety and efficacy data and to provide further directions [31]. At about the same time, the clinical trials in Africa and Asia were stopped and the manufacturer withdrew the product. The situation and conclusions of the meeting were captured in an updated WHO position paper published in 2003 [32], with a strong recommendation that future vaccines should be concomitantly tested in industrialized and developing countries.

In 2004 [33], SAGE was briefed on the development of additional RV candidates, one of which, developed by GlaxoSmithKline Biologicals (GSK), had been licensed in Mexico. This, and another candidate, developed by Merck & Co (Merck), had undergone large-scale clinical trials in Latin America, as well as in the industrialized world, with strong evidence of safety and efficacy [34,35]. Additional trials were being initiated in Asia and Africa, which would also investigate the question of potential RV interference with oral polio vaccine.

Considering data assembled by the PATH Rotavirus Vaccine Programme, SAGE in November, 2005 [17]requested, more information from Africa and Asia where the disease burden was very high, as well as evidence of potential financing mechanisms. SAGE also recognized the importance of post-marketing surveillance to assess safety in real-life situations, and supported previous WHO recommendations for obtaining information on co-administration of RV with routine childhood vaccines. SAGE noted the need to implement strong communication strategies to prevent misconceptions regarding the efficacy of rotavirus vaccines to prevent all childhood diarrhoea. Approaches to this were explored by PATH [36].

SAGE then issued a limited recommendation, captured in a 2007 position paper [37], for use of RV in national immunization programmes in regions where vaccine efficacy data suggested a significant public health impact, and where appropriate infrastructure and financing mechanisms were available. The position paper stated that global inclusion of rotavirus vaccines into national immunization programmes would not be recommended "until the full potential of the current rotavirus vaccines has been confirmed in all regions of the world, in particular in Asia and Africa."

This limited position contrasts with the global recommendation for PCV given above, because of the lack of definitive efficacy data in some parts of the world. The 2007 RV position paper also pointed out the need for surveillance systems which would assess vaccine impact on disease and monitor for increased incidence of intussusception [38]; expressed concern about the timing of receipt of the first dose, which should be well before the age of 12 weeks, after which intussusception is more likely to occur; and advocated consideration of cost-effectiveness and affordability of the vaccine and its financial and operational impact on the immunization delivery system and on immunization practice.

In November, 2008, SAGE considered preliminary clinical trial results from Africa and results from cost-effectiveness studies. These results had been considered at two expert meetings, and were provided to SAGE in November 2008 [39]. The conclusions from these two meetings were that there appear to be no safety concerns; there is no evidence for interference with oral polio vaccine; and South African trial efficacy is equivalent to that found in Latin America. SAGE considered the Asian and African data set at its April 2009 meeting and recently published its current position, which is a recommendation for global use [40].

In summary, for the new RVs licensed in 2004, SAGE received data in that year demonstrating safety and efficacy in a limited number of countries. It requested more information, including a wider range of data, and consideration of vaccine supply strategies, advocacy and logistics issues. Lacking safety and efficacy data in Asia and Africa, the position paper contained a limited recommendation for use, only in countries where safety and efficacy information was available, and thus a global use recommendation was delayed.

Fourteen countries, of which eight are in Latin America, are using the vaccines as of late 2008, three countries have been approved for financial support, and an additional 11 are projected to apply soon [41,42]. Uniquely, the vaccine was introduced into Latin American countries in parallel with introduction into developed countries.

##### Human papillomavirus vaccine

The first time this vaccine was presented to SAGE was at its November 2005 meeting [17]. SAGE noted the potential communications challenges that might exist for a vaccine against a sexually transmitted disease, and suggested the need to study the impact of population screening in conjunction with the vaccine, and the financial implications of such a course.

SAGE was updated in subsequent meetings on the global burden of cervical cancer and the available data on safety, efficacy, and immunogenicity of the two candidate vaccines [43]. In addition, work on cost-effectiveness and vaccine delivery options was described, along with ongoing research on alternative schedules, delivery costs, and acceptability. The particular areas of SAGE focus were that the use of the vaccine was likely to bring great benefits in settings with high cervical cancer burden, especially in countries where screening programmes were limited or non-existent; that data on long-term duration of protection would be important in planning vaccine delivery strategies, especially the need for a booster dose; that the issue of delivery strategies would be critical, as immunizing adolescents entails an expansion into a new target group; and it noted the current high price of the vaccine licensed in industrialized countries [44].

At its June 2007 meeting, the IVB GACVS had determined that there were no outstanding safety issues with use of the vaccine [45]. Work to clarify the issues and to develop candidate recommendations was performed by an HPV Expert Advisory Group (HEAG), which was later reconstituted as the HPV Advisory Committee (HVAC) [46].

Candidate recommendations were presented to SAGE in November 2008 [39], and at that time SAGE recommended that HPV be included in all national immunization programs, provided that (1) prevention of cervical cancer and other HPV-related diseases is a public health priority; (2) an introduction program is nationally feasible; (3) sustainable financing is available; and (4) cost-effectiveness of an HPV intervention is considered. A WHO position paper was published in April 2009 [47].

The work on HPV is an important model for the evolution of policy positions for malaria vaccines, in light of the use of an Expert Advisory Group which included experts in immunization, reproductive health, and cancer to put the requisite data into a background paper and to draft potential positions on vaccine use. A malaria vaccine policy recommendation is also likely to require participants representing a wide group of disciplines. Also, as noted above, key issues for the SAGE were financing and cost-effectiveness issues as well as safety and efficacy.

In the case of HPV, there has been only a two-and-a-half year gap between licensure and a global recommendation. Although the two HPV products are widely licensed, the vaccine has not yet been introduced into developing countries. This likely will happen through GAVI processes in the poorest countries.

#### Policies on malaria interventions

This analysis originally considered four malaria interventions, including artemisinin-based combination therapy, insecticide-treated nets, intermittent preventive treatment in pregnancy, and IPTi. Due to changes in the malaria policy-making structure, the processes used and the completeness of data available for review were not deemed appropriate to this discussion for the first three interventions; thus only IPTi is considered.

IPTi uses the national immunization program to distribute the anti-malarial sulphadoxine-pyrimethamine (SP) to children during routine immunization contacts. An analysis of the decision-making process associated with the development of an IPTi policy is instructive in that it has incorporated the policy development processes of both the GMP STAG and the IVB SAGE.

Promising research results using IPTi from Tanzania [48-50], Ghana [51], and Mozambique [52] are available, and thus the GMP, along with the IPTi Consortium, outlined a policy development plan for this intervention [53], which included consideration by the relevant technical expert groups to GMP (namely, the TEG on Chemotherapy of Malaria and the STAG) and by SAGE.

Because of the SAGE policy of "horizon scanning", the use of IPTi had already been considered in April 2006 [54]. At that time SAGE was presented with the following information [8]: malaria disease burden in sub-Saharan Africa; pooled data from five safety and efficacy studies in sub-Saharan Africa; pooled data from three studies on impact of the intervention on serology *vs *routine infant vaccines; data from two cost-effectiveness studies; information on drug resistance; and one year of implementation experience.

SAGE, after reviewing data from these five trials, and noting declining efficacy, likely related to SP resistance, asked for more safety and efficacy data in relevant populations, including longer term follow-up to rule out rebound (i.e. increasing disease burden later in life) and to indicate efficacy if the putative immunization schedule were not rigorously followed. SAGE also requested more information on the impact of IPTi on alternative malaria control interventions already in force, as well as the usefulness of such a strategy in areas with only seasonal malaria transmission.

The GMP's TEG on Chemotherapy of Malaria met in Geneva in October 2006 [55]. The group reviewed 11 studies on IPTi conducted in Africa in areas of high malaria endemicity. There was a consistent decline in protective efficacy from studies of IPTi with SP over the period 1999 to 2005. Furthermore, a review of the safety data from unpublished studies suggested a small, not statistically significant, increase in severe dermatological reactions (Stevens-Johnson Syndrome) in the SP arms. The results suggesting protection from anaemia were not supported in the further studies. No evidence was found of rebound in malaria mortality during 5 to 12 months after the intervention. The TEG found IPTi with SP to be a promising intervention in settings where there is a malaria burden for infants; where there are rigorous systems to monitor adverse events and drug resistance; where IPTi intervention does not detract from current efforts to scale up other malaria control strategies; where the effectiveness of IPTi is monitored within the context of other existing control interventions; and where the medicines used do not compromise current and future medicines for curative malaria treatment. The TEG called for more research on the question of SP resistance, optimal dosing, alternative drugs, and impact on other malaria control strategies.

After reviewing the available data and some new studies that appeared in 2007 [56,57], WHO reconvened the TEG in October 2007. The TEG was asked to consider efficacy issues in the context of increasing SP resistance, as well as to review possible dermatological reactions. Due to safety concerns related to a few cases of rebound of malaria susceptibility that might indicate issues if IPTi were to be administered to healthy children in a population with declining malaria disease and increasing resistance to SP, and because of the uncertainty related to the optimal dose and timing of administration, the TEG concluded that it could not recommend general deployment of SP in an IPTi strategy [58]. The TEG suggested additional research in these areas and agreed to reconsider the issue in 2008.

In 2008, a review of the evidence based on six trials was published by the Institute of Medicine [59]. The group recommended that there be an independent technical review of the study data and analyses included in the pooled analysis; that IPTi with SP first be implemented in areas of high transmission to obtain the highest public health impact; that public health authorities monitor evidence on changes in SP resistance; and that initial implementation be supplemented with strong efforts to monitor safety, effectiveness, cost-effectiveness, acceptability, sustainability and logistical practicality, to help develop guidelines for larger-scale implementation.

The TEG then considered the information in April 2009 [60], reviewing six studies with SP, and concluded that previous safety concerns were mitigated, that the benefits of IPTi with SP in areas where SP remained effective were upheld as providing 30% overall protection against clinical malaria episodes for approximately 35 days following administration of each dose. The TEG found there remained uncertainties on the potential impact of IPTi with SP on incidence of severe malaria or malaria mortality especially at low levels of malaria transmission. Recommendations were that use of IPTi with SP should be focused on moderate to high transmission areas where parasite resistance to SP is not high, and where implementation would not detract from other malaria preventive measures. Such implementation should be accompanied by continuous surveillance efforts.

Subsequently, SAGE reviewed these recommendations at its October 2009 meeting [61]. SAGE was reassured that experience with IPTi with SP administered with the second and third doses of DTP and with measles seen in pilot projects in Africa did not have a detrimental effect, that EPI monitoring has been successfully adapted to IPTi impact, and that the intervention was generally well accepted by health workers and caregivers. SAGE therefore endorsed the use of IPTi with SP with immunization and recommended that programs implementing this should regularly monitor and evaluate the impact on immunization services and performance; that impact on serological response to pneumococcal and rotavirus vaccines should be undertaken; and that development of a liquid SP formulation is needed.

## Discussion and evaluation

### Lessons learned from previous policy decisions

In the case studies considered above, issues besides safety and efficacy in the relevant target populations have been important and have delayed policy recommendations. In the case of Hib, the major sources of delay were information on disease burden in specific populations and supply issues, despite a wealth of safety and efficacy information. Attention to disease burden and cost-effectiveness in a variety of populations allowed a more rapid global use recommendation to be made for PCV. For RV, the major issue regarding a delay in a obtaining a global recommendation was the time to accumulate data on safety and efficacy in different parts of the world. However, other issues have been addressed and RV is already introduced in a number of countries in Latin America; countries in other regions are poised to introduce this intervention as well. For HPV, as well as for PCV, the use of an expert group to develop background documents and draft candidate recommendations seems to have accelerated the process. It has been consistently noted that the SAGE has requested information on disease burden, cost-effectiveness, impact on other interventions, supply, and financing issues in addition to safety and efficacy data as part of its process for developing policy recommendations.

In the case of IPTi, the need for further safety and efficacy data had been the major factor in delaying a policy recommendation for both the SAGE and the TEG. However, both groups have considered other factors, such as impact on other interventions and disease characteristics, and impact on EPI through pilot projects.

### Data needs for malaria vaccine policy positions

Based on the above analysis for the five interventions considered, it is expected that the SAGE will carefully scrutinize vaccine safety data (relying on its GACVS); require efficacy data from a range of epidemiological settings; consider implementation issues such as local disease burden data, ability of surveillance infrastructure to measure impact, distribution strategies, and alignment of product profile with health systems; carefully consider the impact of an intervention on other prevention and treatment interventions in the future (it has already done so recently for HPV); and consider issues such as vaccine supply, price, availability, regulatory status, and cost-effectiveness. SAGE can be anticipated to have an iterative process of establishing an initial position, then modifying its recommendations over time. Its position is likely to focus on regions where efficacy data are available, given that malaria epidemiology varies in different parts of the world. Its position will become more comprehensive after documentation of successful implementation in one or more developing countries.

One particular issue that may be a focus for SAGE is the selection of the regulatory pathway(s). If the same vaccine could be approved for similar use for the military and traveller markets as for the developing world, then a typical regulatory approach could be followed, with appropriate ongoing pharmacovigilance. However, if a vaccine were developed only for use in, for example, high disease burden areas in Africa, then a specific regulatory pathway would have to be selected which would ensure the gathering of an adequate database on safety in that population, oversight by a regulatory authority able to meet the requirements for WHO prequalification, and considerations for phase 4 studies and enhanced safety surveillance in that population post approval.

SAGE will likely consider the impact of vaccines with varying ranges of efficacy on other malaria control interventions, look at the proposed delivery schedule in the context of national policies, and request evidence that vaccine use will not result in displacing the disease burden to another susceptible age range. However, it is important to point out that SAGE has been reconstituted only recently to its current format, and thus its decision-making process is in evolution.

SAGE did consider the current status of the RTS,S/AS01 candidate malaria vaccine in its October 2009 meeting [61], including the phase 2 trial data and phase 3 trial design. Some of the issues noted above were presented as having already been considered by an "Initiative for Vaccine Research-Global Malaria Programme Joint Technical Expert Groups on Malaria Vaccines Entering Pivotal Phase III Trials and Beyond."

Table 2 includes information based upon the previous analyses, which is likely to be needed to inform policy positions on malaria vaccines. This information could be the target of further refinement by WHO, and in the meantime provides indications of the data that those working on malaria vaccines should strive to address during the development process.

**Table 2:** Draft list of key data considerations information malaria vaccine decision-making based on documentation and WHO interviews

Safety and efficacy in relevant populations	Safety An acceptable safety profile Freedom from "rebound" effect, that is, enhancing disease incidence in target groups following use: needs follow-up population monitoring Positive evaluation from WHO GACVS No significant adverse impact on other malaria prevention and treatment strategies (i.e. increasing adverse events from another product) or on response to concomitantly administered vaccines Safety evaluated in immunologically compromised groups, e.g. HIV-infected
	
	Efficacy Acceptable level of reduction of disease-related morbidity and/or mortality in target populations Efficacy demonstrated in different malaria endemicity settings Delivery schedules, dosing and administration route feasible and consistent with burden of disease in target countries
	
Implications for costs and population health	Supply, financing, and cost-effectiveness issues Availability of product under the regulatory oversight of a fully functional regulatory authority and/or prequalification Available supply related to anticipated demand Affordability Means of monitoring impact to feed into cost-effectiveness assessment Prospects for competitive vaccine market
	**Impact on other public health interventions** Vaccine delivery strategies to reach desired target groups (e.g., catch-up immunization where relevant) Impact of vaccine use on compliance with other interventions, e.g. ITN Community perception of given malaria vaccine products given their likely characteristics Impact of the vaccine demonstrated in the context of other malaria control strategies

**Localization of data**	**Local applications of the intervention** Evidence sufficient for local decision making available to the appropriate in-country groups (such as Immunization Advisory Committee, Interagency Coordinating Committee, etc), including, as relevant, national stakeholders and decision makers and key partners Ability to deliver vaccine through local cold chains Specific evidence for unique epidemiological situations available, if applicable Information from demonstration projects available particularly where new target groups or specific product acceptance issues are involved

### The policy-making process for a malaria vaccine

There are a number of possible models, which could be used for malaria vaccines, based on the policy-making experience described above. Three different models were considered by the authors. The first, based upon the HPV model, is a time-limited group representing TEG and a sub-group of SAGE, which could then report back to SAGE and STAG. The second potential model, drawing on the RV experience, is to use or modify the membership of the existing MALVAC committee, a group advisory to IVB considering research and development (R&D) approaches to a malaria vaccine [62]. The committee's focus on R&D may limit its capacity to provide guidance on policy recommendations for vaccine implementation.

The third approach is modelled upon the IPTi process using the appropriate TEG to provide SAGE and STAG with recommendations. However, no member of the currently established TEG on Chemotherapy of Malaria has expertise in immunology or vaccinology, and thus the group would not be able to effectively review data on immune response, impact on response to other vaccines, schedule information, logistics and delivery, and supply issues. A modification of this is to convene a new TEG under joint WHO immunization and malaria leadership, which would draw upon expertise from both disciplines. This in fact has been done, as noted above, convening its first meeting in mid-2009.

## Conclusions

With the above as background, several conclusions can be drawn:

1. Although efficacy and safety in the relevant target population, including displacement of disease to another susceptible age range, are crucial components of any policy development process, additional concerns may also be important in the formulation of policy positions. These include issues related to costs and population health, such as supply and demand issues, financing issues, cost-effectiveness, the impact of use on other interventions, and issues specific to the local situation, such as distribution and cold chain considerations. Specific issues unique to a malaria vaccine listed in Table 2 will need consideration.

2. All efforts should be made to document the use of a malaria vaccine in the context of other malaria treatment and prevention interventions, and to rigorously document the impact.

3. In the analyses above, SAGE issued initial positions, while asking that demonstration projects or pilot introduction studies be done in some cases (e.g. HPV), where a new target population or a specific product communication issue is involved. Such studies could assist to refine the WHO policy recommendation.

4. For a product with a complex policy development process like a malaria vaccine, a specially convened working group may be useful to assist both GMP and IVB policy groups.

5. A strong recommendation in a WHO position paper is important for efficient decision-making on use by developing countries, as seen, for example, in the case of Hib. The vaccine interventions studied in this analysis demonstrate a very positive shortening of the time required by WHO to issue such recommendations.

This paper has taken the unique step of trying to identify the data and processes required at the WHO policy level for the introduction of a new intervention. While the focus has been on SAGE and STAG processes for malaria vaccines, it is critical to also find the correct balance of input from national and regional levels. Organizations can work in advance to address data requirements; however, lessons from historical precedents, as developed in this paper, or specific inputs of WHO, are required to guide this work. Developing data in advance and further clarifying policy processes will allow policy-making bodies to continue their trend towards more efficient decisions, while also considering the full range of essential information. Shaving months and years off of the policy-making process will ultimately mean that life-saving interventions can get to those in need much sooner than has been seen historically.

## Competing interests

The authors declare that they have no competing interests.

## Authors' contributions

JM performed the analysis and drafted the paper. VC participated in the analysis and contributed to the drafting of the paper. JC participated in the initial planning for the analysis, participated in the analysis and contributed to the drafting of the paper. AB conceived the study, contributed to the analysis and to the drafting, and provided overall guidance and oversight. All authors have read and approved the final manuscript.
